# Abnormal cerebellar network and effective connectivity in sudden and long-term sensorineural hearing loss

**DOI:** 10.3389/fnagi.2022.964349

**Published:** 2022-08-11

**Authors:** Jin-Chao Hua, Xiao-Min Xu, Zhen-Gui Xu, Yuan Xue, Jin-Jing Xu, Jing-Hua Hu, Yuanqing Wu, Yu-Chen Chen

**Affiliations:** ^1^Department of Otolaryngology, Nanjing Pukou Central Hospital, Pukou Branch Hospital of Jiangsu Province Hospital, Nanjing, China; ^2^Department of Radiology, Nanjing First Hospital, Nanjing Medical University, Nanjing, China; ^3^Department of Otolaryngology, Nanjing First Hospital, Nanjing Medical University, Nanjing, China

**Keywords:** cerebellar network, sudden sensorineural hearing loss, long term sensorineural hearing loss, independent component analysis, effective connectivity

## Abstract

Sudden sensorineural hearing loss (SSNHL) is a common otology emergency and some SSNHL will develop into a long-term hearing loss (LSNHL). However, whether SSNHL and LSNHL have similar psychiatric patterns remains unknown, as well as the neural substrates. Increasing evidence has proved that the cerebellar network plays a vital role in hearing, cognition processing, and emotion control. Thus, we recruited 20 right SSNHL (RSSNHL), 20 right LSNHL (RLSNHL), and 24 well-matched healthy controls to explore the cerebellar patterns among the three groups. Every participant underwent pure tone audiometry tests, neuropsychological evaluations, and MRI scanning. Independent component analysis (ICA) was carried out on the MRI data and the cerebellar network was extracted. Granger causality analysis (GCA) was conducted using the significant cerebellar region as a seed. Pearson’s correlation analysis was computed between imaging characteristics and clinical features. ICA found the effect of group on right cerebellum lobule V for the cerebellar network. Then, we found decreased outflow from right cerebellum lobule V to right middle orbitofrontal cortex, inferior frontal gyrus, anterior cingulate cortex, superior temporal gyrus, and dorsal lateral prefrontal cortex in RSSNHL group in GCA analysis. No significance was found in RLSNHL subjects. Additionally, the RSSNHL group showed increased effective connectivity from the right middle frontal gyrus (MFG) and the RLSNHL group showed increased effective connectivity from the right insula and temporal pole to the right cerebellum lobule V. Moreover, connections between right cerebellum lobule V and mean time series of the cerebellar network was negatively correlated with anxiety score in RSSNHL and negatively correlated with depression scores in RLSNHL. Effective connectivity from right MFG to right cerebellum lobule V could predict anxiety status in RSSNHL subjects. Our results may prove potential imaging biomarkers and treatment targets for hearing loss in future work.

## Introduction

Sensorineural hearing loss (SNHL) is the most common sensory deficit with the death of hair cells, spiral ganglion, or auditory nerve fibers (Géléoc and Holt, 2014). Estimates suggested that by the year 2050, it will affect up to 900 million individuals worldwide. Sudden sensorineural hearing loss (SSNHL) was defined by De Kleyn (De Kleyn, 1944) as sudden onset of SNHL of >30 dB in three contiguous frequencies for three days or less, accompanied by tinnitus and vertigo sometimes. SSNHL can generally improve within a matter of days (Schreiber et al., 2010) while SSNHL patients who cannot recover within 2 weeks will likely develop into long-term SSNHL (LSNHL; Cho et al., 2022).

The effects of hearing impairment can not only be peripheral but also be central. Impaired ability of communication after auditory deprivation would lead to a reduced quality of life, resulting in annoyance, frustration, depression, and social isolation (Mick et al., 2014; Kamil and Lin, 2015; Force et al., 2021). Research on congenital deafness and presbycusis indicated that hearing loss (HL) caused inattention and increased the risk of dementia in later life (Evans, 2006; Lin et al., 2013). However, whether SSNHL and LSNHL have similar psychiatric patterns remains unknown.

There is increasing awareness that the cerebellum is not only associated with motor function but also with cognition processing and emotion control (Schmahmann and Caplan, 2006; Moreno-Rius, 2018). The cerebellum was involved in the underlying neural circuit of anxiety, fear, deficits of executive control, spatial cognition, and memory (Buckner, 2013; Otsuka et al., 2016; Llano et al., 2021). In addition, the cerebellum is considered to play a much border role in sensory and perceptual aspects (Baumann et al., 2015). Except for the primary auditory cortex, the cerebellum has been documented as the second active region during auditory tasks (Petacchi et al., 2005). Human and animal studies also found its participation in hearing impairments, including tinnitus, hyperacusis, and HL (Velluti and Crispino, 1979; Chen et al., 2015). Manganese-enhanced magnetic resonance imaging demonstrated and evaluated spontaneous activity of the cerebellum in rats with tinnitus, counting it as a tinnitus generator (Brozoski et al., 2007; Zhang et al., 2018). Neuroimaging study in unilateral HL noted enhanced and weakened connectivity between the cerebellar network and other systems (Zhang et al., 2018). Our previous finding has implicated the role of the cerebellum in LSNHL (Xu et al., 2019). But no one has focused on the local cerebellar network and effective connectivity in different duration of SNHL.

The present study was designed to explore: (1) alterations of the cerebellar network following right SSNHL (RSSNHL) and right LSNHL (RLSNHL); (2) causal relationship between cerebellum and other brain regions. To address it, we used independent component analysis (ICA) to extract the cerebellar network, which has been proved as a robust tool for identifying and reconstructing temporally-coherent, spatially-independent networks based on group connectivity rather than individual level (Jafri et al., 2008; van Belle et al., 2014). Compared to the seed-based method, ICA has been proven to avoid *a-priori* seed selection as well as to reduce the heterogeneity of the cerebellar network pattern, thus allowing for unbiased exploration of the association between the cerebellar network and cognitive function (Dacosta-Aguayo et al., 2014). ICA also helps to separate signal fluctuations in RSNs from each other and automatically captures the entire cerebellar network as a single major component. Furthermore, Granger causality analysis (GCA) was conducted to determine whether the cerebellar time series was useful for forecasting other brain areas since it was widely applied due to its ability of characterizing the flow information of diverse sources of data (Stokes and Purdon, 2017). We assumed that: (1) RSSNHL and RLSNHL patients would show aberrant intrinsic connectivity within the cerebellar network relative to healthy controls, and (2) effective connectivity between the cerebellum and other cortical regions would be further detected, which was correlated with specific neuropsychological status of SNHL.

## Materials and Methods

### Subjects and clinical assessment

We recruited 20 RSSNHL (12 males and eight females, mean age of 51.5 + 7.0 years), 20 RLSNHL (eight males and 12 females, mean age of 53.1 + 12.3 years), and 24 well-matched healthy controls (12 males and 12 females, 56.5 + 6.9 years) from the local community and E.N.T. department of our hospital *via* advertisements. A professional audiologist diagnosed SNHL with pure tone audiometry (PTA) in six frequencies (0.25, 0.5, 1, 2, 4, and 8 kHz) using a GSI-61 audiometer, as well as with otoscopy to exclude middle ear infection, tympanic membrane perforation, and cerumen.

Subjects who met the following criteria were included in our study: (1) 20–70 years old; (2) right-handedness; (3) had an education level of 6 years at least; (4) postlingual deafness; (5) hearing threshold of right ear >30 dB in at least three frequencies; (6) hearing threshold of left ear <25 dB in all six frequencies. Individuals who: (1) had pulsatile tinnitus, conductive deafness, Meniere’s disease, otosclerosis, head tumors, and MRI contraindications; (2) had a history of head trauma, stroke, psychiatric illnesses, Alzheimer’s disease, and neurosurgery; (3) suffered from drugs or alcohol addiction were excluded.

Every participant underwent neurological scales and MRI scanning respectively. We computed mini-mental state examination (MMSE), symbol digit modalities test (SDMT), and auditory verbal learning test (AVLT) to assess cognition status; self-rating anxiety scale (SAS), and Hamilton depression scale (HAMD) to evaluate anxiety and depression. Subjects with MMSE scores <26 were removed from our study.

### MRI data acquisition

All subjects were scanned on 3.0 Tesla MRI with an 8-channel head coil (Ingenia, Philips Medical Systems, Netherlands). Everyone was asked to lie quietly with eyes closed, stay awake, and avoid thinking about special things during acquisition. We used a foam pad to minimize head motion and earplugs to attenuate scanner noise. Firstly, structure images were acquired with a T1-weighted 3D spoiled gradient-echo sequence: repetition time (TR) = 8.1 ms, echo time (TE) = 3.7 ms, flip angle (FA) = 8°, field of view (FOV) = 256 mm × 256 mm, matrix = 256 × 256, 170 slices, slice thickness = 1.0 mm. Then, functional images based on BOLD were acquired axially using a gradient-echo-planar (EPI) sequence with next parameters: TR/TE = 2,000/30 ms, FA = 90°, FOV = 240 mm × 240 mm, matrix = 64 × 64, 36 slices, slice thickness = 4 mm.

### Data preprocessing

Image preprocessing was carried out using Data Processing and Analysis of Brain Imaging toolbox (DPARBI^1^) and Statistical Parametric Mapping software (SPM 12^2^). We discarded the first 10 volumes of functional images for signal equilibrium and used the remaining 230 volumes for analysis: slice timing, realignment, co-registration with the anatomical scan, segment into gray matter (GM), white matter (WM), and cerebrospinal fluid (CSF), and normalization into Montreal Neurological Institute (MNI) template (resampling to 3 × 3 × 3 mm^3^). After that, all functional images were smoothed using a 6 mm full-width half-maximum (FWHM) Gaussian kernel. None of the 64 subjects were excluded because of head motion >2.0 mm in maximum displacement or >2.0° rotation in any direction. Twenty-four various variances, including motion parameters, white matter, and CSF signals were removed from signals *via* linear regression.

### Independent component analysis

Before ICA analysis, we performed voxel-based morphometry (VBM) to generate GM volume of each subject using VBM- DARTEL method (Umeda et al., 2015), and no significant difference was found among groups. Age, gender, education level, and GM volume were added as covariates in the subsequent statistical analysis to control these confounders. Group spatial ICA was conducted to extract the cerebellar network using GIFT software package^3^ based on Matlab^4^. All preprocessed functional images were decomposed into 30 components using the infomax algorithm following repeated 100 times analysis with ICASSO (Himberg et al., 2004). The intensity values of connectivity within each independent component were converted to z-scores to reflect the degree to which the time series of a given voxel correlated with the mean time series of its corresponding component. One-sample t-test with false discovery rate (FDR) correction (*p* < 0.01) was applied to define the cerebellar network according to a previous study (Huang et al., 2018), with the distinct peak of power spectrum at low-frequency (<0.1 Hz) range, and spatial pattern and periodic temporal fluctuation.

### Granger causality analysis

In this study, we used GCA analysis to describe the causal relationship between the reference time series of ROI and the time series of other brain regions based on REST software^5^. As a definition from Granger in the field of economics (Granger, 1969), if the given time series of x could predict the time series of y, we thought y must have a causal influence on x. Here, the time series of right cerebellum lobule V was defined as x, and the time series of other brain areas were defined as y. The linear direct effect of x on y (Fx→y) and the linear effect of y on x (Fy→x) were calculated voxel by voxel across the whole brain. Thus, each subject had two Granger causality maps and these maps were converted to z-values using Fisher’s r to z transformation (Zx→y and Zy→x) to improve the normality.

### Statistical analysis

The demographic and neuropsychological data were performed by SPSS software (Version 21.0, Chicago). The chi-square test was applied to evaluate gender distribution. Data were expressed as mean + standard deviation, and a one-way analysis of variance (ANOVA) was conducted on all continuous variables. A *post-hoc* test was computed to find the difference between patients and Controls, and the significant threshold was set at *p* < 0.05.

Functional data (including VBM, ICA, and GCA) were analyzed using REST software. We compared the difference among RSSNHL, LSSNHL, and Control groups using one-way ANOVA for multiple comparisons, Family-wise error (FWE) correction with *p* < 0.05 was of significance. *Post-hoc* test with Bonferroni correction was performed to explore the intergroup difference within a mask of ANOVA results. After that, we extracted the signals of significant results and computed Pearson’s correlation to analyze the relationships between functional images and neuropsychological test (*p* < 0.05).

## Results

### Demographic and neuropsychological data

Totally, 64 subjects were included in our research, including 20 RSSNHL patients, 20 RLSNHL patients, and 24 Controls. There was no significance in terms of age, gender, education level, and hearing threshold of the left ear. The hearing ability of the right ear in both RSSNHL and RLSNHL groups was much worse than Controls (*p* < 0.001). From the perspective of neuropsychological tests, SAS score, SDMT score, AVLT tests of immediate and delayed memory were of significance while there was no significant difference in MMSE and HAMD scales (Table 1). *Post-hoc* analysis using Dunnett t-test (two-sided) found that SDMT (*p* < 0.001), AVLT-2 (*p* = 0.001), AVLT-3 (*p* < 0.001), AVLT-5 min (*p* < 0.001) and AVLT-20 min (*p* = 0.016) performance in RLSNHL group were worse than Controls, while SAS scores in RSSNHL group were higher than Controls (*p* < 0.001). We could conclude from the above data that different durations of HL led to various neuropsychological deficits as RSSNHL induced anxiety and RLSNHL resulted in impaired cognition.

**Table 1:** Demographic information, auditory ability, and neuropsychological features of RSSNHL, RLSNHL, and Controls.

**Characteristics**	**RSSNHL (*n* = 20)**	**RLSNHL (*n* = 20)**	**Controls (*n* = 24)**	**P value**
**Demographic information**
Age (year)	51.5 ± 7.0	53.1 ± 12.3	56.5 ± 6.9	0.163
Gender (M/F)	12:8	8:12	12:12	0.449
Education (year)	14.3 ± 4.0	12.5 ± 3.2	12.2 ± 2.9	0.102
Duration (day)	8.2 ± 6.2	365*(7.4 ± 6.1)	-	-
**Audiology test**
Right ear (dB HL)	76.1 ± 23.6	76.1 ± 24.1	18.5 ± 2.3	<0.001
Left ear (dB HL)	20.1 ± 5.9	20.0 ± 5.0	17.3 ± 2.1	0.074
**Neuropsychological tests**
MMSE (score)	29.8 ± 0.5	29.6 ± 0.6	29.9 ± 0.4	0.128
SDMT (score)	47.2 ± 13.6	36.3 ± 13.1	47.0 ± 10.1	0.003
AVLT 1 (n)	4.4 ± 1.3	3.8 ± 1.3	4.4 ± 1.1	0.136
AVLT 2 (n)	6.8 ± 2.0	5 ± 1.4	6.5 ± 1.8	0.003
AVLT 3 (n)	7.7 ± 1.7	5.7 ± 0.9	7.1 ± 1.2	<0.001
AVLT 5 min (n)	7.2 ± 1.9	5.3 ± 1.0	6.9 ± 1.1	<0.001
AVLT 20 min (n)	7.0 ± 2.0	5.2 ± 1.2	7.0 ± 1.1	<0.001
SAS (score)	50.3 ± 10.7	37.6 ± 7.9	36.7 ± 12.9	<0.001
HAMD (score)	7.5 ± 2.9	6.4 ± 4.5	5.2 ± 4.1	0.166

Data are expressed as Mean ± standard deviation. * means multiply. RSSNHL, right sudden sensorineural hearing loss; RLSNHL, right long-term sensorineural hearing loss. MMSE, Mini-mental state examination; SDMT, symbol digit modalities test; AVLT, auditory verbal learning test; SAS, self-rating anxiety scale; HAMD, Hamilton depression scale.

### Cerebellar network

**Figure 1:**
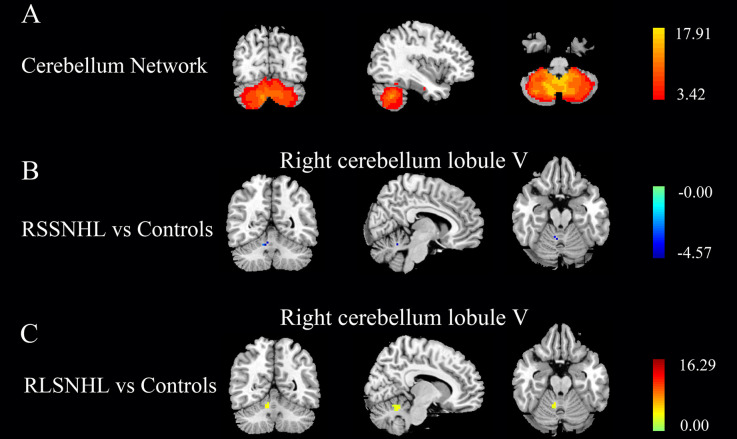
Independent components analysis among RSSNHL, RLSNHL, and Controls. **(A)** Spatial maps of identified cerebellar network. **(B)** Difference of cerebellar network between RSSNHL and Controls. **(C)** Difference of cerebellar network between RLSNHL and Controls. The significant p was set at <0.05 with family-wise error correction. RSSNHL, right sudden sensorineural hearing loss; RLSNHL, right long-term sensorineural hearing loss.

Firstly, VBM analysis failed to find significance among the three groups. Figure 1A was the cerebellar network we extracted using the ICA method based on functional images. ANOVA revealed an effect of group on right cerebellum lobule V for the cerebellar network. As suggested by *post-hoc* analysis, we found a weak functional connectivity (FC) between right cerebellum lobule V and mean time series of the cerebellar network in the RSSNHL group, but enhanced connections in the RLSNHL group, compared to Controls (Figures 1B,C; *p* < 0.05/3).

### Effective connectivity

**Figure 2:**
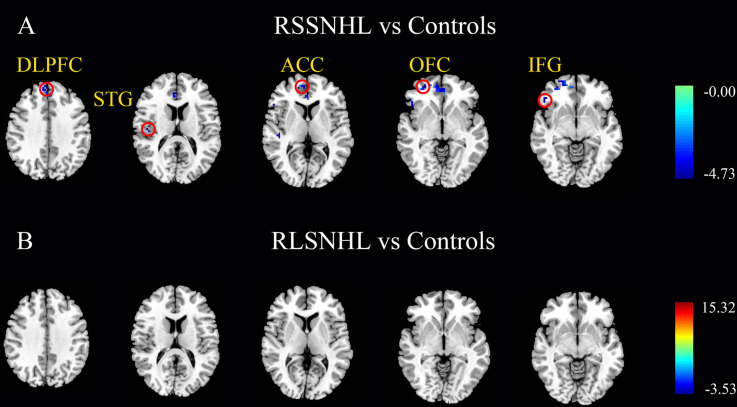
Effective connectivity from cerebellum lobule V among RSSNHL, RLSNHL, and Controls. **(A)** Significant regions between RSSNHL and Controls. **(B)** No significance between RLSNHL and Controls. The significant p was set at <0.05 with family-wise error correction. The region with the significant difference among the three groups is shown with color. OFC, middle orbitofrontal cortex; IFG, inferior frontal gyrus; ACC, anterior cingulate cortex; STG, superior temporal gyrus; DLPFC, dorsal lateral prefrontal cortex; RSSNHL, right sudden sensorineural hearing loss; RLSNHL, right long-term sensorineural hearing loss.

As indicated in the ICA analysis, we chose the right cerebellum lobule V as a seed for GCA analysis. Compared with Controls, between-group analysis, we found decreased outflow from right cerebellum lobule V to right middle orbitofrontal cortex (OFC), inferior frontal gyrus (IFG), anterior cingulate cortex (ACC), superior temporal gyrus (STG), and dorsal lateral prefrontal cortex (DLPFC) in RSSNHL group (Figure 2A, Table 2). No significance was found in RLSNHL subjects (Figure 2B, Table 2).

**Table 2:** Effective connections from right cerebellum lobe V among RSSNHL, RLSNHL, and Controls.

**Brain region**	**BA**	**MNI coordinate x, y, z (mm)**	**Peak T value**	**Cluster size**
**RSSNHL vs. Controls**
R middle orbitofrontal cortex	11	27, 54, −3	−4.7221	35
R inferior frontal gyrus	47	48, 33, −6	−4.6796	18
R anterior cingulate cortex	10	6, 54, 6	−4.7126	94
R superior temporal cortex	48	48, −18, 15	−4.6981	20
R dorsal lateral prefrontal cortex	9	3, 51, 36	−4.5584	17
**RLSNHL vs. Controls**	**No significance**

BA, Brodmann area; MNI, Montreal Neurological Institute coordinates; RSSNHL, right sudden sensorineural hearing loss; RLSNHL, right long-term sensorineural hearing loss.

**Figure 3:**
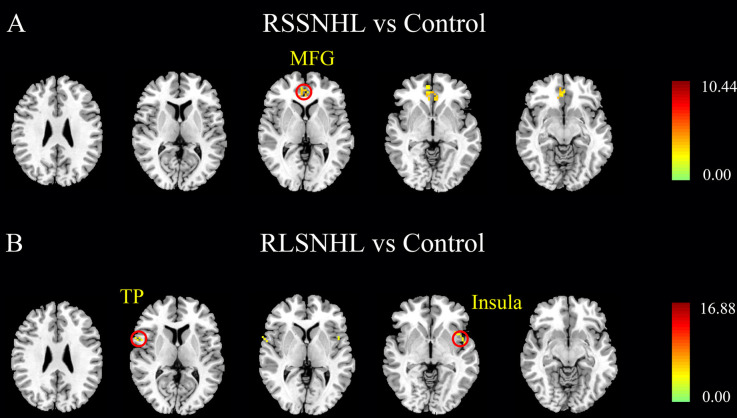
Effective connectivity to the cerebellum lobule V among RSSNHL, RLSNHL, and Controls. **(A)** Significant regions between RSSNHL and Controls. **(B)** Significant regions between RLSNHL and Controls. The significant p was set at <0.05 with family-wise error correction. The region with the significant difference among the three groups is shown with color. MFG, middle frontal gyrus; TP, temporal pole; RSSNHL, right sudden sensorineural hearing loss; RLSNHL, right long-term sensorineural hearing loss.

Besides, we visually observed that the RSSNHL group showed increased effective connectivity from the right middle frontal gyrus (MFG) and the RLSNHL group showed increased effective connectivity from the right insula and temporal pole (TP) to the right cerebellum lobule V (Figures 3A,B, Table 3). The significant *p* was set at <0.05 with FWE correction.

**Table 3:** Effective connections to right cerebellum lobe V among RSSNHL, RLSNHL, and Controls.

Brain region	BA	MNI coordinate x, y, z (mm)	Peak T value	Cluster size
**RSSNHL vs. Controls**
R middle frontal gyrus	10	6, 48, −9	4.1917	83
**RLSNHL vs. Controls**
L insula	48	−48, 6, 0	4.557	11
R temporal pole	48	63, 6, 3	4.4012	13

BA, Brodmann area; MNI, Montreal Neurological Institute coordinates; RSSNHL, right sudden sensorineural hearing loss; RLSNHL, right long-term sensorineural hearing loss.

### Correlation analysis

**Figure 4:**
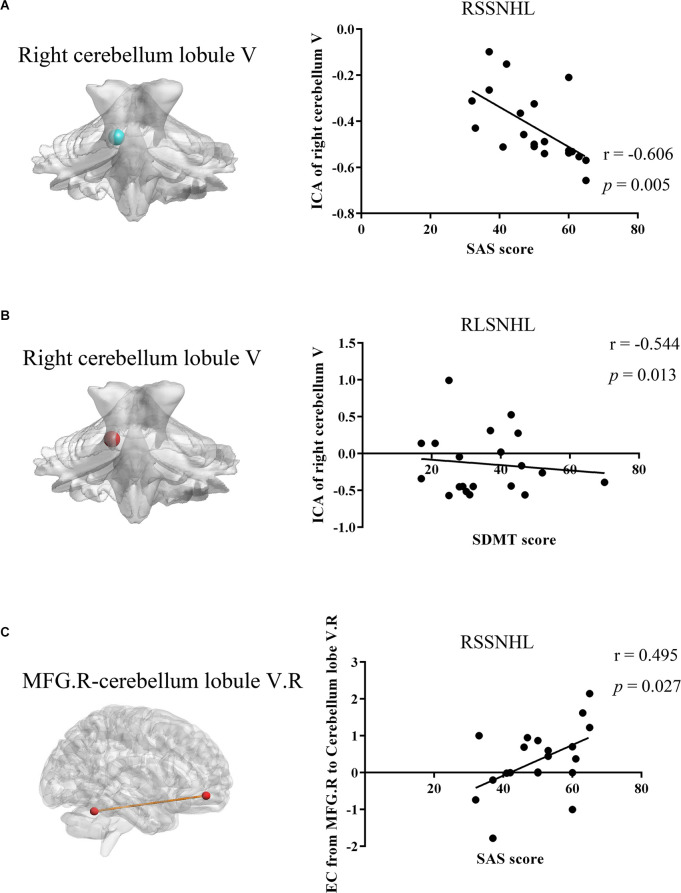
Relationships between MRI imaging properties and clinical features in RSSNHL and RLSNHL. **(A)** Decreased functional connectivity between the right cerebellum lobule V and mean time series of cerebellar network in RSSNHL group was negatively correlated with SAS score (*r* = −0.606, *p* = 0.005). **(B)** Increased connections between the right cerebellum lobule V and mean time series of the cerebellar network in RLSNHL group showed negative correlation with SDMT performance (*r* = −0.544, *p* = 0.013). **(C)** Effective connectivity from right MFG to the right cerebellum lobule V could predict anxiety status in RSSNHL subjects (*r* = 0.495, *p* = 0.027). MRI, magnetic resonance imaging; ICA, independent components analysis; SAS, self-rating anxiety scale; SDMT, Hamilton depression scale; MFG, middle frontal gyrus; RSSNHL, right sudden sensorineural hearing loss; RLSNHL, right long-term sensorineural hearing loss.

Pearson’s correlation analysis revealed that decreased FC between right cerebellum lobule V and mean time series of the cerebellar network in the RSSNHL group was negatively correlated with SAS score (*r* = −0.606, *p* = 0.005, Figure 4A). And the increased connections between right cerebellum lobule V and the mean time series of the cerebellar network in the RLSNHL group showed a negative correlation with SDMT performance (*r* = −0.544, *p* = 0.013, Figure 4B). Moreover, the effective connectivity from right MFG to right cerebellum lobule V could predict anxiety status in RSSNHL subjects (*r* = 0.495, *p* = 0.027, Figure 4C).

## Discussion

In the present study, we identified an altered cerebellar network using ICA measurement and abnormal casual flow of right cerebellum lobule V in acute and long-term right SNHL, along with anxiety in sudden SNHL and cognitive impairments in long-term SNHL. Weakened inter-connectivity in the cerebellar network and enhanced inflow from MFG to right cerebellum lobule V in acute SNHL were negatively and positively correlated with SAS scores respectively. Increased inter-connection in the cerebellar network in long-term SNHL showed a negative correlation with cognition ability. Taken together, we speculated that SNHL disrupted functional integration of the cerebellar network and the cerebellar network had distinct functions in sudden and long-term SNHL, contributing to SNHL-induced neuropsychological deficits.

Previous nationwide population-based cohort studies in Taiwan (Tseng et al., 2016) and Korea (Kim et al., 2018) demonstrated an increased risk of affective disorders following SSNHL, including anxiety and depression, which were consistent with our findings. One possible explanation is the stress mechanism, as acoustic trauma could cause anxiety and transient oxidative stress in an animal model (Zheng et al., 2014). Moreover, Masuda et al. (2012) proposed some inflammatory biomarkers related to system stress in patients with SSNHL, like neutrophil counts and natural killer cell activity. Second, epidemiologic studies indicated the bidirectional relationships between anxiety disorders and SSNHL (Stein, 2009). Third, as an otology emergency, SSNHL with pervasive tinnitus often developed psychological perturbations and tinnitus individuals also had a high prevalence of anxiety (Muhlmeier et al., 2016). Patients whose symptoms cannot alleviate will then develop into long-term auditory deprivation and performed worse in SDMT and AVLT. Hearing impairment in the elderly has been found to contribute to cognitive dysfunction (Strutt et al., 2022) and many neurodegeneration diseases dementia commonly involve sensory systems, including auditory (Hardy et al., 2016). Additionally, mice with HL exhibited working and recognition memory deficits, as well as increased p-tau and lipofuscin expression in the hippocampus (Park et al., 2018), which could be evidence of HL-related dementia.

However, we failed to find the disparity in GM among these three groups. VBM analysis in unilateral SSNHL demonstrated GM alterations of contralateral auditory cortical morphology (Fan et al., 2015) and non-auditory brain areas (Yang et al., 2014). Boyen et al. (2013) observed GM increases in STG, while Alfandari et al. (2018) did not find any brain volume changes in the primary auditory cortex and WM underneath it, but increased GM in angular gyrus and decreased WM in the fusiform gyrus in long-term HL. Furthermore, neuroimaging studies uncovered that tinnitus and vertigo also had various effects on GM and WM across the whole brain (Meyer et al., 2016; Scott-Wittenborn et al., 2017). Altogether, these inconsistencies of structure MRI in SNHL were possibly because of heterogeneity of participants, duration of diseases, residual hearing, accompanying symptoms, and different psychological conditions.

Our ICA results indicated an abnormal role of the right cerebellum lobule V among these three groups. As part of the anterior lobule, cerebellum lobule V has been confirmed to function in sensory (Diano et al., 2016), somatosensory (O’Reilly et al., 2010), and sensorimotor patterns (Kawabata et al., 2022). In the present study, we found decreased cerebellar FC in RSSNHL and increased cerebellar FC in RLSNHL. As the cerebellum lobule V is involved in the anterior lobe (sensorimotor cerebellum), sudden hearing deprivation led to weakened neural activity in the acute period. The possible mechanism of enhanced synchronicity in cerebellum lobule V may be neural plasticity. By manipulating the activity of the cerebellum lobule V, Chao et al. found its correlation with social process, anxiety behaviors, and cognition in animal research (Chao et al., 2021). Moreover, we found a significant negative correlation between lobule V and SAS/SDMT behaviors. FC analysis of cortico-cerebellar circuits proved the interactions of STG and lobule V, providing evidence of participation of lobule V in auditory processing (Stoodley, 2012). And the lobule V was also engaged in language processing and reading, which need the cooperation of normal hearing (McDermott et al., 2003).

OFC locates within the frontal lobe and is a part of the limbic system, which is involved in cognition and emotion. It receives projections from the temporal lobe and has been proved that higher listening effort was associated with thinning and grey matter loss in the OFC in age-related hearing loss (Rosemann and Thiel, 2020). Song et al. (2013) found decreased connectivity between OFC and the auditory cortex in patients with single-sided deafness. In our results, we also found the abnormalities of other subregions in the frontal lobe, including IFG, DLPFC, and MFG. In previous research (Xu et al., 2019), we disclosed the weakened couplings between the cerebellum and other brain areas in long-term bilateral SNHL, including IFG and MFG, which may support part of the present findings here. DLPFC has been recognized as a key node in cognitive processing and executive function. Moreover, it may influence emotional reactivity by altering higher-order perceptual attention systems, because it doesn’t receive direct projection from emotion generators (Luan et al., 2019). However, [18F] fluorodeoxyglucose (FDG)-PET of elderly patients with hearing aids detected increased metabolism in the prefrontal gyrus and the cingulum (Verger et al., 2017). This inconsistence may be due to the use of hearing aids and neural plasticity after hearing recovery.

Besides, the RSSNHL group showed weakened connections from the cerebellum lobule V to ACC. It is known that ACC has connections to both the emotional limbic system and the cognitive prefrontal cortex anatomically. Conversely, a relative increase in FDG uptake was found in ACC in RSSNHL patients within 72 h of onset, as well as a decrease in FDG uptake in the insula (Micarelli et al., 2017). Heterogeneity of duration could be the possible reason since the duration of RSSNHL in our study is 8.2 ± 6.2 days. What is more, RLSNHL showed enhanced connectivity from the insula to cerebellum lobule V. The insula plays an important role in multimodal sensory processing, audio-visual integration, and cognition (Fitzhugh and Pa, 2022). We need to do more work to figure out the mechanism of recruitment of insula in acute and long-term SNHL.

Our study explored underlying neural substrates of different duration of hearing impairments and improved better understanding of the impact of auditory disability, providing potential therapeutic targets for future research. It still has several limitations in our research. First, the relatively small sample of subjects might affect the statistical power, and generalizability of results. And we could not rule out some accompanying symptoms, such as tinnitus or vertigo. Second, various etiological factors of sudden and long-term hearing loss would have different effects on brain functions, so that larger datasets need to be collected to control these confounding factors. Third, we did not pay much attention to the frequencies of hearing loss, which likely influence neural activities. Finally, we focused on the cerebellum lobule V using the ICA method, further work needs to be done to find vital roles of other cerebellar subregions.

## Conclusion

To conclude, the current study revealed the key role of the cerebellar network among RSSNHL, RLSNHL, and Controls, especially the cerebellum lobule V. Additionally, causal connections between the cerebellar network and other brain areas were also demonstrated, as well as correlations with neural deficits, disclosing the neuropathological mechanism underlying acute and long-term hearing deprivation.

## Data Availability Statement

The original contributions presented in the study are included in the article, further inquiries can be directed to the corresponding authors.

## Ethics Statement

The studies involving human participants were reviewed and approved by Research Ethics Committee of the Nanjing Medical University and in accordance with the Declaration of Helsinki. The patients/participants provided their written informed consent to participate in this study.

## Author Contributions

J-CH and X-MX collected the MRI data, performed the analysis, and wrote this manuscript. Z-GX helped with data analysis and discussion. YW, J-JX, and J-HH contributed to recruitment of subjects and data analysis. Y-CC and YX designed this study and helped with the writing of this manuscript. All authors contributed to the article and approved the submitted version.

## Funding

This work was supported by Doctoral Program of Entrepreneurship and Innovation in Jiangsu Province (No. JSSCBS20211544), Xinghuo Talent Program of Nanjing First Hospital, Nanjing Special Fund for Health Science and Technology Development (No. YKK21133), Natural Science Foundation of Jiangsu Province (No. BK20211008), and Medical Science and Technology Development Foundation of Nanjing Department of Health (No. ZKX20037).

## Footnotes

^1^ http://rfmri.org/dparbi

^2^ http://www.fil.ion.ucl.ac.uk/spm

^3^ http://icatb.sourceforge.net/

^4^ https://www.mathworks.com/

^5^ http://www.restfmri.net
